# Midline Sacral Fractures: Review of the Literature

**DOI:** 10.51894/001c.38909

**Published:** 2023-12-05

**Authors:** Ivan Bandovic, Benjamin Diedring, Adrian Olson, Sean Tan, Marek Denisuk, Dexter Powell, Benjamin Best

**Affiliations:** 1 Orthopaedic Surgery Ascension Macomb Oakland Hospital; 2 Orthopaedic Surgery Ascension Macomb-Oakland Hospital; 3 Osteopathic Medicine New York Institute of Osteopathic Medicine; 4 Orthopaedic Surgery Ascension Macomb-Oakland Hospital,; 5 Orthopaedic Surgery Ascension Providence Hospital; 6 Orthopaedic Surgery Ascension St. John Hospital

**Keywords:** Midline sacral fracture, vertical sacral fracture, sacrum, pelvic ring injury

## Abstract

**INTRODUCTION:**

Sacral fractures are an important consideration in high-energy traumas associated with injuries to the pelvic ring that confer much of pelvic stability. A midline longitudinal sacral fracture (MLS) is a relatively rare fracture pattern, with only 23 cases of MLS fractures reported in the literature to date. This systematic review evaluates overall mechanisms of MLS injury, associated injuries, complications, management, and fracture prognosis.

**METHODS:**

A 1952-2021 PubMed literature search yielded 11 publications reporting the outcomes of a total of 23 MLS fracture cases.

**RESULTS:**

Of the 23 MLS patients, 15 (65%) were male and eight (35%) were female, with an average age of 37.25. Ten (43.5%) MLS fractures occurred during motor vehicle collisions and eight (34.7%) because of motorcycle accidents. The most common pelvic ring injuries associated with MLS were pubic symphysis diastasis (n = 12, 57%) and pubic ramus fractures (n = 11, 48%). Patients most frequently suffered intra-pelvic organ dysfunction such as sexual dysfunction or bowel/bladder/urethral injuries. Fractures were treated both operatively or non-operatively and generally showed clinical meaningful resolution at 10 weeks post-injury.

**CONCLUSIONS:**

MLS injuries most often occur in high-energy trauma due to motor vehicle or motorcycle accidents as well as crush injuries, leg splitting, direct perineal/perianal impacts. Pre-trauma sacral abnormalities could be potentially predisposing factors correlated with MLS fractures. Careful review of x-rays and CT scans may help reveal MLS fractures, which can go initially undiagnosed. Operative and nonoperative management strategies includes bedrest, transsacral transiliac screw, decompressive laminotomies, and/or pelvic external fixation. The outcomes reported to date have been generally favorable, with most patients healing at approximately 10 weeks. Keywords: Midline sacral fracture; vertical sacral fracture; sacrum; pelvic ring injury

## INTRODUCTION

Sacral fractures are an important consideration in high-energy traumas (e.g., motor-vehicle accidents, falls from height, high-force impact) associated with pelvic ring injuries since the sacrum confers much pelvic stability.^1^ The magnitude and direction of force resulting in pelvic ring injury patterns can be categorized based on the injury mechanism causing the fracture whether that be hemipelvis external rotation, lateral compression or vertical shear.^2^

The most commonly cited classification system for pelvic ring injuries, described in 1986 by Young et al, identifies pelvic injury patterns as either a vertical shear (VS), a lateral compression (LC) or anterior posterior compression (APC), which the latter two can also be further sub-categorized into LC or APC Types I through III.^2^ Although most pelvis ring injuries arise from high-energy injuries resulting in multiple fractures and ligamentous damage requiring surgery, Type I injuries often arise from lower energy injuries in the elderly, and can frequently treated non-operatively.^2^

Although sacral fractures often go undiagnosed on initial imaging, clinicians should hold suspicion for a sacral fracture in patients who sustained high energy trauma with posterior sacral pain.^1^ Anterior-posterior (AP), inlet, and outlet radiographs of the pelvis, as well as Computerized Tomography (CT) scans can aid in diagnosis. If not immediately available, medical stabilization and transfer to a facility with an orthopedic trauma specialist is also recommended.

In their landmark 1988 paper, Denis et al. retrospectively reviewed 236 sacral fractures. The authors categorized sacral fractures into three zones, based on the medial extent of the fracture. Zone I fractures were located lateral to the neuroforamina involving the lateral sacral ala bone. Zone II fractures were located through the region of the sacral neuroforamina, and Zone III fractures were primarily located medial to the neuroforamina.^3^

**Figure 1.**
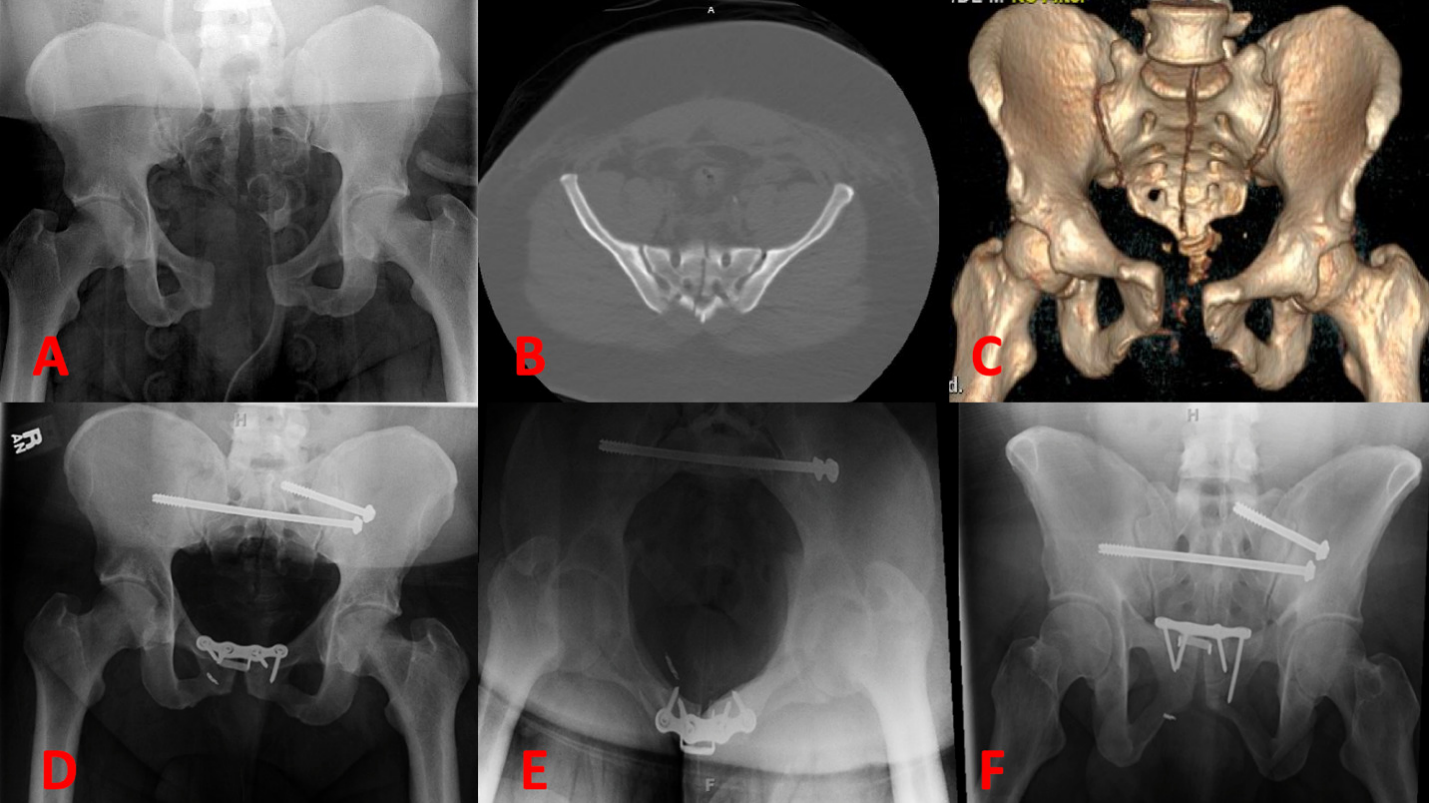
A complete anterior to posterior midline longitudinal sacral fracture as seen on (A) Anterior-Posterior (AP) Radiograph, (B) Axial cut of CT Pelvis (bone window), (C) 3D reconstruction, and post-operative (D) AP, (E) inlet, and (F) outlet radiographs.

Of note, Zone III fractures can include transverse type fractures or U-shaped fractures, whose fracture lines may cross multiple zones. The Dennis group found 118 (50%) of sacral fractures in Zone I, 81 (34.3%) fractures in Zone II, and 37 (16%) fractures in Zone III. Midline longitudinal sacral (MLS) fractures are very rare fractures isolated to Zone III, and the subject of discussion in this review (Figures 1A-C).

### Example Patient

For example, the authors of this paper treated a 41-year-old male (as a 23^rd^ case) with a body mass index of 52. who had sustained an MLS fracture from a motorcycle accident. The patient additionally suffered a sacroiliac joint dislocation, and 3.7 cm pubic symphysis diastasis. The patient was treated with sacroiliac screw, transsacral transiliac screw, and open reduction internal fixation (ORIF) of the pubic symphysis (Fig. 1D-F).

The patient was placed on non-weight bearing precautions for 12 weeks and began ambulating without pelvic pain at 12 weeks post-operatively as is typical for most MLS fracture patients. Of note, the hardware at the pubic symphysis did show signs of early loosening, however his sacral hardware and pelvic ring remained stabled at one-year follow-up.

### Purpose of Review

The aim of this 1952-2021 systematic review was to investigate the literature to date concerning MLS fractures, and formulate a report of demographic, associated bony and soft tissue pelvic injury, complications, management, and prognosis of published cases.

## METHODS

Authors IB and ST performed an extensive literature search on the topic of sacral fractures using the PubMed database. Search terms included “sacral fracture”, “sacrum fracture”, “Denis Zone III”, “longitudinal”, “midline”, “vertical”, and “mid sagittal”. Data were collected from 11 studies reporting on cases of midline vertical sacral fractures, including the 23^rd^ patient highlighted in this report.

## SUMMARY OF LITERATURE

A total of 11 previously published studies reporting on a total of 22 patients were included in this review (the 23^rd^ patient described above was included in the descriptive statistics). The first reported case of an MLS fracture was published by Wiesel et al. in 1979 and was treated with bedrest for six weeks.^4^ Subsequent reported cases similarly showed success with non-operative management of these fractures, including external stabilization with a pelvic sling, protected weight bearing with crutches, and oral analgesics.^5,6^ Carter el al. surgically stabilized the pubic symphysis diastasis, however felt the sacrum to be stable and treated it non-operatively with crutches. The same authors also reported the first case of an MLS fracture with spina bifida occulta, whose abnormal sacral anatomy may have contributed to the pathomechanics of the MLS fracture pattern.^6^

In 1996, Ebraheim et al. also reported a patient who was a pygopagus twin, a conjoined twin fused at the sacrum, who was separated at birth.^7^ It was only noted intraoperatively that the patient had a previous procedure, and it is plausible that this post-surgical defect after twin separation could have created a stress riser in the sacrum. Soon after Ebraheim’s first report, the same authors reported a subsequent case series of four patients, aged 16-59, who all sustained MLS fractures through motor vehicle collisions.^8^ They successfully treated two patients with transsacral transiliac screws for fixation of their MLS fractures.

The same 1996 authors noted two patients had spina bifida occulta & a low lying dural sac to S1. Kaneko et al. reported a 2001 case of a 47-year-old male who was successfully treated with a pelvic external fixator (i.e., an external scaffold pinned to the bony pelvis to provide stability) for a complex pelvic ring fracture involving an MLS fracture.^9^ A 2003 case series by Bellabarabara et al. described a series of 10 MLS fractures, all from APC type pelvic ring injuries.^10^ Eight patients were treated with ORIF of associated pubic symphysis diastasis, and one with a pelvic external fixator. They did not report ORIF of the sacrum in any patients, or the use of the pelvic external fixator for the sacral fracture. Patients required an average of 10 weeks to heal, and reported no neurological deficits associated with sacral nerve root injury, no residual pain or gait disturbances, no loss of bowel or bladder function, no loss of perianal sensation or sphincter tone, and no radicular or sensory motor deficits in the bilateral lower extremities; However, three patients had complaints of sexual dysfunction, which was attributed to blunt trauma to the perineum.^10^

In 2005, Harma et al. reported a case of a 20-year-old male who was hit in the back by a rolling boulder.^11^ The patient returned to weightbearing at 12 weeks after ORIF of his pubic symphysis, and pelvic external fixator for his pubic rami fractures and sacroiliac joint dislocation. The clinician group did not believe additional posterior fixation of the MLS fracture would bring additional benefit as the fracture did not extend caudally past the S1 vertebrae.

In 2017, Vijayan et al. reported a case of a 55-year-old male who was in a road traffic accident, who successfully underwent ORIF for pubic symphysis diastasis, and an anterior pelvic external fixator for MLS fracture.^12^ Finally, O’Neill et al. in 2019 reported the case of a 67-year-old male who was involved in a motorcycle versus automobile accident and sustained an MLS fracture treated with two transsacral transiliac screws.^13^

## RESULTS

In summary, a total of 23 patient cases were identified for this review. The gender affiliation and ages of these cases are summarized in Table 1.

**Table 1.** MLS Fracture Patient Characteristics (N = 23)

**Gender Affiliation**	n (%)
Male^4,6,7,9,10,12,13^	15 (65%)
Female^5,6,8,10^	8 (35)

**Average age (years)**	38.5 ± 11.7
Male^4,6,7,9,10,12,13^	39.2 ± 10.5
Female^5,6,8,10^	37.25± 14.4

NOTE: Bellabarbara et al.^9^ did not disclose the age of each patient, but rather the average age of all patients (i.e., 36 years).

Table 2 summarizes MLS fracture details including mechanism of injury, associated pelvic ring injuries, other associated injuries.

**Table 2.** MLS Fracture Details (N = 23)

	N (%)
**Mechanism of injury**	
Motor Vehicle Collision^7–10,12^	10 (46%)
Motorcycle Accident^10,13^	8 (35%)
Other *^4–6,10,11^	5 (23%)
	
**Associated pelvic ring injuries**	
Pubic Symphysis diastasis^4–6,10–13^	13 (57%)
Pubic Ramus fracture^7–11^	11 (48%)
Ilium fracture^7^	1 (5%)
SacroIliac Joint Dislocation	1 (5%)
	
**Associated other injuries/deficits**	
Sexual Dysfunction/Impotence^10,13^	4 (18%)
Bladder injury^10^	3 (14%)
Urethral injury^10^	3 (14%)
Bowel/Rectal Injury^6–8,10^	3 (14%)
Sensory deficits^5,7^	2 (9%)
Vaginal wall injury**^5^	1 (4%)

*Category includes fall from height, leg split, bucked off horse onto saddle, crush injury, pedestrian vs auto

**Out of eight female patients

Table 3 summarizes the management of MLS fractures in the literature.

**Table 3.** Treatment and Outcomes of MLS fractures*

	N (%)
**Non-operative** ^4–6,10–12^	16
Bedrest^4,5,7,11^	4 (25%)
Protected Weight Bearing^6^	1 (6%)
Weight Bearing as Tolerated^10^	10 (62.5%)
Not specified^12^	1 (6%)
**Operative** ^7,8,11,13^	7
Transsacral transiliac screw^7,13^	4 (57%)
Decompressive laminectomy^7,8^	4 (57%)
External-Fixator^11^	1 (7%)

**Outcomes**	
**Non-operative** ^4–6,10–12^	16
Healing at 6 weeks^4–6,8^	4 (25%)
Healing at 10 weeks^10^	10 (62.5%)
Healing at 12 weeks^12^	1 (6%)
Healing at 6 months^12^	1 (6%)
**Operative** ^7,8,11,13^	7
Transsacral transiliac screw^7,13^	4 (57%)
Healing at 6 weeks^7^	2
Healing at 10 weeks^13^	2
Decompressive laminectomy^7,8^	4 (57%)
Healing at 12 weeks^7^	1
Not specified^7,8^	3
External-Fixator^11^	1 (14%)
Healing at 12 weeks^11^	1

*Management is specifically for the MLS fracture. Patients with pelvic ring injuries, i.e. pubic diastasis & MLS fracture who were only treated with pubic symphysis ORIF were considered non-operative.

It is important for readers to note that the descriptive statistics for treatment only include MLS fractures, not other pelvic ring injuries. Patients treated only with ORIF of the pubic symphysis were considered by the authors to be managed “non-operatively” for their MLS fractures.

### Recovery Patterns

Of the 23 reviewed patients, 16 (70%) patients were treated nonoperatively and successfully healed their injuries for between six weeks to six months.^4–6,10–12^ These patients were treated with either bedrest (25%),^4,5,7,11^ protected weight bearing (6%),^6^ or weight bearing as tolerated (62.5%).^10^ Only one (6%) report did not indicate weight bearing status in the non-operatively treated patient.^12^ Of the patients who were treated nonoperatively, four patients (25%) healed without complications within six weeks,^4–6,8^ 10 (62.5%) healed without complications within 10 weeks.^10^ One patient (6%) took 12 weeks to heal.^12^ Only one patient (6%) took six months to heal with no complications.^12^

Of the 23 patients, seven patients (30%) were treated operatively using transsacral transiliac screws, decompressive laminectomies, and external fixation, alone or in combination.^7,8,11,13^ Four patients (57%) were treated with transsacral transiliac screws,^7,13^ four patients (57%) with decompressive laminectomies alone,^7,8^ and one patient (14%) with an external fixator alone.^11^ Two patients (28.5%) received both decompressive laminectomies and transsacral transiliac screws. The patients treated operatively healed with no complications within 6 - 12 weeks.^7,8,11,13^

### Post-Injury Sequelae

Blunt trauma to the pelvis resulted in a variety of pelvic organ dysfunction. Four patients (18.2%) complained of sexual dysfunction and/or impotence.^10,13^ Three patients (13.6%) sustained bladder injury,^10^ three patients (13.6%) with urethral injury,^10^ and three patients (13.6%) who sustained bowel/rectal injuries (e.g., rupture, perforation, decreased rectal tone).^6–8,10^

A smaller percentage of these patients suffered from other sequelae. Two patients (9.1%) had a lower extremity sensory deficit or perianal paresthesia.^5,7^ One female patient (12.5% of female patients) suffered from a vaginal wall injury.^5^

## DISCUSSION

In summary, the literature to date has characterized MLS fractures as being largely associated with APC injury secondary to a high-energy mechanism without significant neurological injury. Eighteen of 23 reviewed cases have been secondary to a MVC or motorcycle accident. There have been no reported cases of MLS fractures with lateral compression type pelvic injuries.

MLS fractures, like many sacral fractures, may be missed on plain AP pelvis x rays due to the superimposed curvature of the sacrum, or slight malrotation of the pelvis, masking the fracture.^1^ Therefore, it is crucial for general surgery /trauma team, emergency physicians, and orthopedic specialists to further evaluate the sacrum with all pelvic ring injury patterns.^11^ A CT scan as well as pelvic outlet/inlet radiograph may be required to evaluate the sacrum and sacral spinal canal.^12^

Blunt trauma to soft tissues of the lower abdomen and genitourinary system made up most reviewed MLS-associated injuries. Blunt trauma to the perineum and pelvis can cause neuropraxia to the nerves supplying the bowel, bladder, or genitalia, potentially resulting in sexual dysfunction, incontinence, and urinary retention soon after injury.^6,7,9,11,13^ Since long-term follow-up findings were not generally reported in the selected studies, it is unknown how long these deficits persisted.

The authors agree with Bellabarba, et. al. that sexual dysfunction after APC injuries may be related to severe pelvic trauma which leads to thrombosis of the vasculature in the perineum causing impotence.^10^ Based on available literature, MLS fractures are most commonly associated with pubic diastasis and/or pubic rami fractures.

It is reasonable to believe that there may have been undiagnosed destabilizing sacral abnormalities in reviewed patient cases which ultimately predisposed them to MLS type fractures in the setting of APC forces. The authors suggest that clinicians consider, investigate, and report underlying sacral abnormalities if they encounter MLS fractures in practice, to further enhance our understanding of these injuries.

Based on these results, the treatment of most sacral fractures has been largely dependent on the extent of patients’ pelvic ring disruption. Although earlier cases were managed with bedrest and pelvic traction, modern treatment including internal fixation is largely based on restoring pelvic ring stability.^14^

Today, there are multiple treatment options for sacral fractures ranging from non-operative, to operative methods including ORIF with plate fixation, percutaneous fixation using transsacral transiliac screws, or pelvic external fixation.^1^ Additionally, sacral decompression may be indicated in settings of sacral nerve compromise. We have concluded that the most desirable treatment for MLS fractures is percutaneous fixation utilizing transsacral transiliac screws, as it has been reported to have successful outcomes in longitudinal sacral fractures with minimal soft-tissue injury.^1,15^

## CONCLUSIONS

Since MLS fractures are quite rare, there remains a paucity of literature to date derived from a heterogenous set of patient cases. It is our hope that this review can help guide future clinicians to provide evidence-based guidelines for treatment of MLS fractures. Operative and nonoperative management includes bedrest, transsacral transiliac screw, decompressive laminotomies, and/or pelvic external fixation. The outcomes reported in the literature are generally favorable, with most patients healing at approximately 10 weeks.

### Conflict of Interest

None
